# Emergency catheter ablation in critical patients

**DOI:** 10.4103/0974-2700.62118

**Published:** 2010

**Authors:** Jürgen Tebbenjohanns, Klaus Rühmkorf

**Affiliations:** Medic Clinik I, Department of Cardiology, Angiology and Intensive Care Medicine, Klinikum Hildesheim, Weinberg 1, 31134 Hildesheim, Germany

**Keywords:** Arrhythmias, catheter ablation, intensive care medicine

## Abstract

Emergency catheter ablation is justified in critical patients with drug-refractory life-threatening arrhythmias. The procedure can be used for ablation of an accessory pathway in preexcitation syndrome with high risk of ventricular fibrillation and in patients with shock due to ischemic cardiomyopathy and incessant ventricular tachycardia. Emergency catheter ablation can also be justified in patients with an electrical storm of the implanted cardioverter-defibrillator or in patients with idiopathic ventricular fibrillation.

## INTRODUCTION

Performance of catheter ablation as an emergency procedure in critical patients is rare. Catheter ablation is indicated in patients with drug-refractory life-threatening arrhythmias. The scenarios discussed below will illustrate the importance of emergency catheter ablation in preventing fatality.

## PREEXCITATION SYNDROME

Sudden cardiac death can occur in patients with Wolff-Parkinson-White (WPW) syndrome due to rapid antegrade conduction of atrial fibrillation over an accessory pathway, with resulting ventricular fibrillation and cardiac arrest.

**Figure 1:**
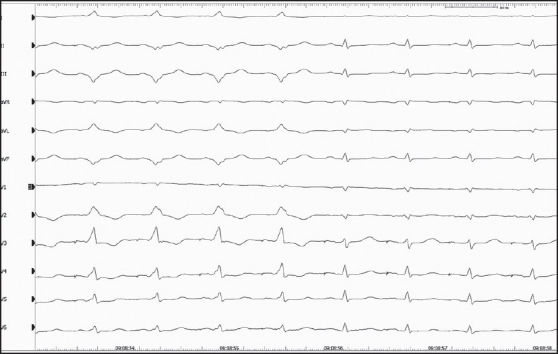
Catheter ablation of a ‘malignant’ accessory pathway in a patient with aborted sudden cardiac death who was successfully resuscitated. During atrial stimulation the first four QRS complexes are broad and the PQ interval is short due to conduction over the accessory pathway. The last four beats show narrow QRS with normal PQ interval after the accessory pathway was successfully ablated

A 31-year-old female patient was resuscitated by her husband after the latter recognized syncope and cessation of breathing. The emergency physician diagnosed ventricular fibrillation and immediate defibrillation resulted in restoration of normal sinus rhythm. The patient was intubated, ventilated, and transferred to the nearest hospital. After 3 days of intensive care therapy, including mild hypothermia, extubation was successfully performed. She had no neurological deficits. A 12-lead surface electrocardiogram (ECG) showed preexcitation syndrome with a suspected accessory pathway at the inferoseptal aspect. The patient was then referred to our hospital for catheter ablation. During mapping of the inferoseptal region of the right atrium close to the coronary sinus ostium, the accessory pathway could be located slightly inside the coronary sinus. Therefore mapping was performed via transaortic access and the accessory pathway was successfully ablated at the atrial aspect of the mitral annulus [Figure 1]. An implantable cardioverter-defibrillator (ICD) was not implanted since in the family no sudden cardiac death occurred, echocardiography was normal, and the 12-lead ECG taken after ablation showed no abnormalities; the recurrence rate in this setting is low.[1] Antz *et al*. reported on 48 patients with WPW syndrome and aborted sudden cardiac death. In all patients, the accessory pathways were successfully ablated and ICD implantation was not performed. During the follow-up period of 5 ± 2 years there was no recurrence of life-threatening arrhythmia or syncope. Therefore catheter ablation of the accessory pathway is the appropriate treatment in this group of patients.

## INCESSANT VENTRICULAR TACHYCARDIAS

Patients with ischemic or nonischemic cardiomyopathy can suffer from so-called incessant ventricular tachycardias (VT). These tachycardias are often slow and therefore incessant and drug refractory. These arrhythmias occur in the setting of severe depressed left ventricular function with large areas of scar tissue. The patients are often on antiarrhythmic drugs (e.g., amiodarone) and sometimes present in cardiogenic shock and have to be treated in intensive care units. The treatment is termination of VT by electrical cardioversion. Additional antiarrhythmic drugs cannot be tolerated as they have negative inotropic action and worsen the patient's condition. In this setting, catheter ablation is the only option for these patients.

**Figure 2:**
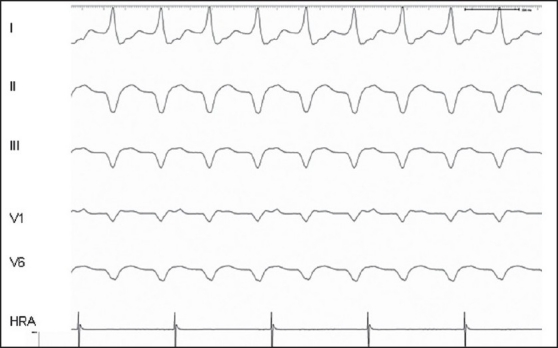
Incessant VT. Bipolar electrogram from the right atrium (HRA) revealed ventricular–atrial (VA) dissociation in a broad QRS complex tachycardia

**Figure 3:**
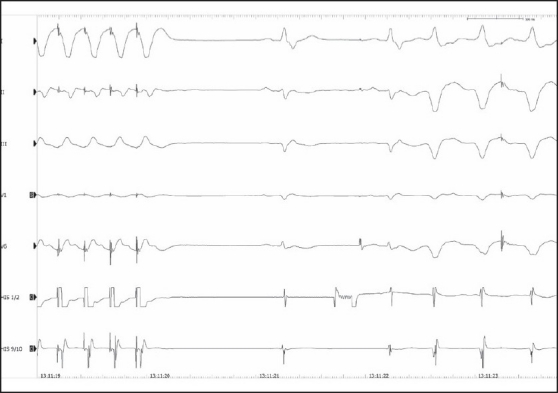
Rapid ventricular pacing during incessant VT. Shown are surface ECG recordings (I, II, III, V1, and V6) and two bipolar electrograms from the right ventricle (RVA). During rapid ventricular pacing the VT terminated, and two beats with normal sinus rhythm can be seen. Immediately after the second sinus rhythm beat the VT started again

A 63-year-old male patient with arrhythmogenic right ventricular cardiomyopathy was referred from another hospital for catheter ablation of incessant VT. Antiarrhythmic drug treatment consisted of a beta-blocker and amiodarone. An ICD had been implanted some years ago after aborted sudden cardiac death. During electrophysiologic study, VT was confirmed [Figure 2]. After rapid ventricular pacing, two beats with normal sinus rhythm were documented [Figure 3]; however, VT started again immediately. During electrogram-based mapping using color-coded CARTO^®^ mapping, the reentry circuit could be identified at the free wall of the right ventricle. Entrainment maneuvers in areas with mid-diastolic potentials were found positive and multiple radiofrequency current applications could terminate the VT. Ablation was started with an 8-mm solid-tip electrode. Later in the procedure, a 4-mm cooled-tip electrode was used. After 36 h in intensive care the VT recovered and a second ablation procedure was performed, with successful termination of VT and restoration of sinus rhythm. The patient recovered quickly, blood pressure returned to normal, and edema and elevated liver enzymes normalized within the next 3 days.

## ELECTRICAL STORM

The so-called electrical storm with multiple ICD discharges due to VT or VF is life threatening, and sudden cardiac death can occur despite the ICD. Amiodarone and β-blockers are almost always given as first-line treatment to terminate VT or VF. In a recent study by Carbucicchio in 95 patients (including 72 with coronary artery disease, 10 with dilative cardiomyopathy, and 13 with arrhythmogenic right ventricular cardiomyopathy), catheter ablation was performed in drug-refractory electrical storm.[2] After one to three ablation procedures VT could be successfully targeted in 85 patients (89%). The electrical storm could be acutely terminated in all patients. After a median of 22 months, 92% of the patients were free of electrical storm and 66% had no recurrence of VT. Eight out of the ten patients who still had inducible VT at the end of the procedure again showed electrical storm of VT during follow-up; four of these patients died suddenly despite adequate ICD shocks. Cardiac mortality was 12% in that study.

**Figure 4:**
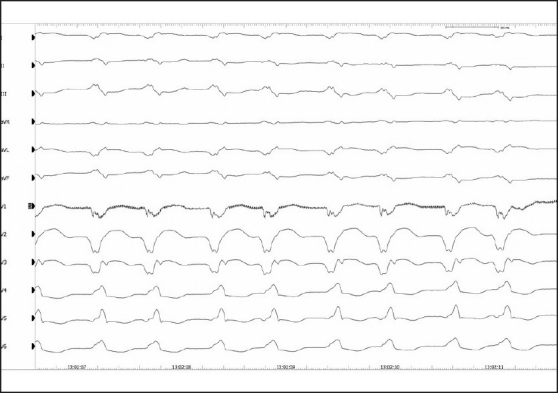
Twelve-lead surface ECG shows a monomorphic VT in a 73-year-old patient with ischemic cardiomyopathy and recurrent ICD discharges

**Figure 5:**
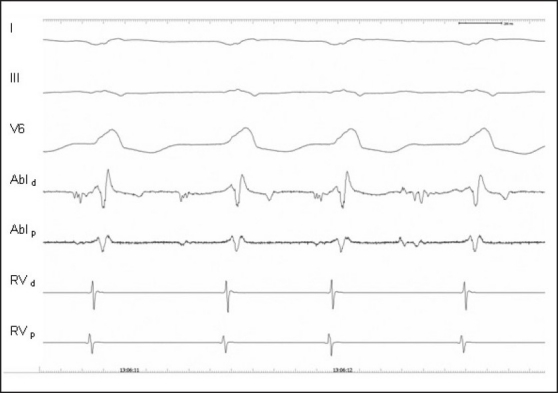
Mapping during VT shows mid-diastolic potentials on the bipolar electrogram at the ablation catheter (AbI). Entrainment stimulation (not shown) confirmed that region as being a critical part of the reentry circuit

**Figure 6:**
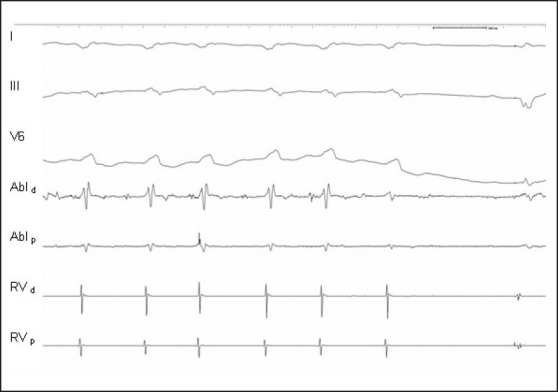
During RF application at that site shown in [figure 5] the VT terminated and normal sinus rhythm was restored

**Figure 7:**
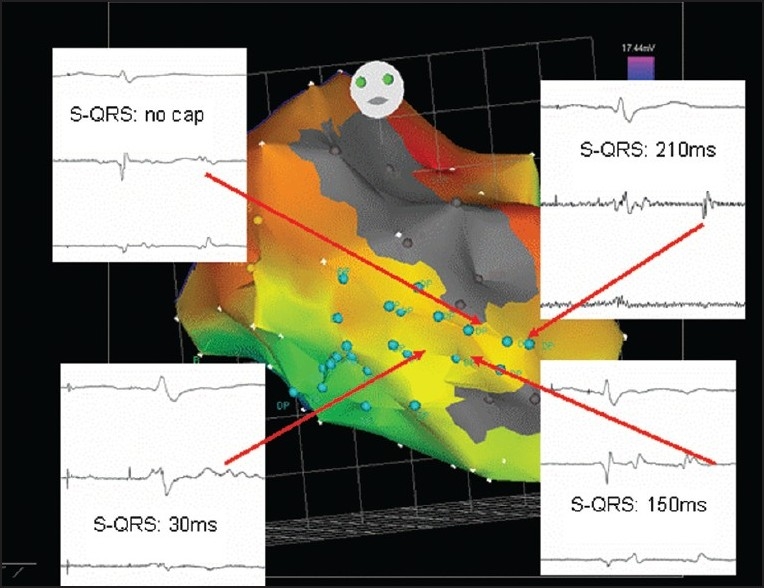
A color-coded CARTO^®^ map of the left ventricle during sinus rhythm is shown in a 71-year-old patient who had multiple ICD discharges for recurrent VT after a large anterior wall infarction. Wide areas of scar tissue are shown (gray color). Isolated myocardial potentials could be recorded between two scar tissue areas (blue points). Stimulation at these sites showed very different stimulus-to-QRS (S-QRS) intervals, indicating different areas within the reentry circuit: entry zone, central zone, or exit zone. An ablation concept could be created by connecting the scar areas with an ablation line to interrupt the reentry circuit of VT

The success rate of catheter ablation for acute treatment of electrical storm in our study is high; similar high rates have also been reported by Schreieck and others. One example of our experience is given in Figures [4–6]. Catheter ablation should be performed early in patients who experience electrical storm. Targeting the VT is also possible during normal sinus rhythm by mapping of fractionated late potentials [Figure 7][3] or the earliest activation of ventricular premature complexes preceding VT onset.[4]

## IDIOPATHIC VENTRICULAR FIBRILLATION

A rather small number of patients in our study had so-called idiopathic VF. Most of them had aborted sudden cardiac death and later had had an ICD implanted. Haissaguerre reported on 27 patients with idiopathic VF without apparent structural heart disease.[5] The unique observation was that a monomorphic ventricular premature complex always served as a trigger of VF. These triggering beats were mapped and ablated at the earliest site of activation. The local electrogram showed rapid deflection on the bipolar recordings that belong to the Purkinje system. The time from local electrogram to surface ventricular complex varied between 10 and 150 ms but was only 11±5 ms during normal sinus rhythm. During a mean follow-up of 2 years in 24 patients (89%) no recurrence of idiopathic VF occurred although no medications were used. Kohsaka and others reported a similar case recently.[6]

## CONCLUSION

From our cases and the results of our interventions we feel that emergency catheter ablation is justified in the following conditions: 1) Wolff-Parkinson-White (WPW) syndrome with risk of sudden cardiac death, 2) incessant VT due to ischemic cardiomyopathic shock, 3) electrical storm in patients with ICDs, and 4) idiopathic VF.
